# Diabetes mellitus in dogs attending UK primary-care practices: frequency, risk factors and survival

**DOI:** 10.1186/s40575-020-00087-7

**Published:** 2020-06-10

**Authors:** Angela M. Heeley, Dan G. O’Neill, Lucy J. Davison, David B. Church, Ellie K. Corless, Dave C. Brodbelt

**Affiliations:** 1 Pathobiology and Population Science, The Royal Veterinary College, Hawkshead Lane, North Mymms, Hatfield, Herts AL9 7TA UK; 2 Clinical Sciences and Services, The Royal Veterinary College, Hawkshead Lane, North Mymms, Hatfield, Herts AL9 7TA UK

**Keywords:** Diabetes mellitus, Risk factors, Survival, Case-control study, Benchmarking, VetCompass

## Abstract

**Background:**

Diabetes mellitus (DM) is an important endocrine disorder of dogs. The objectives of this study were to estimate prevalence and incidence of DM in dogs, and to explore risk factors for DM and the survival of DM cases in primary-care clinics in the UK.

**Results:**

A case-control study nested in the cohort of dogs (*n* = 480,469) aged ≥3 years presenting at 430 VetCompass clinics was used to identify risk factors for DM, using multivariable logistic regression. Overall 409 new and 863 pre-existing DM cases (total 1272) were identified in 2016, giving an apparent annual prevalence of 0.26% (95% confidence interval (CI): 0.25–0.28%), and an annual incidence risk of 0.09% (95%CI: 0.08–0.09%) in dogs aged ≥3 years. Factors associated with increased odds for DM diagnosis were all age categories > 8 years, female entire dogs (odds ratio (OR): 3.03, 95% CI 1.69–5.44, *p* < 0.001) and male neutered dogs (OR: 1.99, 95% CI 1.18–3.34, *p* = 0.010) compared to male entire dogs, Border Terriers (OR: 3.37, 95% CI 1.04–10.98, *p* = 0.043) and West Highland White Terriers (WHWT) (OR: 2.88, 95% CI 1.49–5.56, *p* = 0.002) compared to crossbreeds. Dogs that had received previous glucocorticoid treatment (OR: 2.19, 95% CI 1.02–4.70, *p* = 0.044) and those with concurrent conditions (documented obese, pancreatitis, hyperadrenocorticism) also had increased odds for DM diagnosis.

Cox regression modelling was used to evaluate factors associated with survival in the 409 incident DM cases in 2016. Increased hazard of death following diagnosis of DM was shown in dogs that were ≥ 10 years age, Cocker Spaniels (HR: 2.06, 95% CI 1.06–4.01, *p* = 0.034) compared to crossbreeds, had a blood glucose (BG) level at diagnosis > 40 mmol/L (HR: 2.73, 95% CI 1.35–5.55, *p* = 0.005) compared to < 20 mmol/L at diagnosis, or had received previous glucocorticoid treatment (HR: 1.86, 95% CI 1.21–2.86, *p* = 0.005). Dogs at reduced hazard of death included neutered dogs (HR: 0.58, 95% CI 0.42–0.79, *p* = 0.001), Border Collies (HR: 0.39, 95% CI 0.17–0.87, *p* = 0.022) and those starting insulin treatment (HR: 0.08 95% CI 0.05–0.12, *p* < 0.001).

**Conclusions:**

Certain breeds and concurrent health conditions are associated with an increased risk of DM. In addition to certain signalment factors, a high BG level at diagnosis and prior glucocorticoid treatment were adversely associated with survival of dogs with DM.

## Plain English summary

Diabetes mellitus (DM) is a serious disease that can compromise the welfare of dogs. This study looked at factors associated with the risk of dogs developing DM, and also factors associated with how long they might survive with the disease.

The study compared 409 dogs from UK primary-care practice diagnosed with DM in 2016, with 818 dogs without DM. Dogs that were more likely to be diagnosed with DM included those that were older than 8 years, female dogs that were not neutered, male dogs that were neutered, Border Terriers, West Highland White Terriers (WHWTs), those who had previous been on glucocorticoid (steroid) medication, and those with other health conditions such as obesity, pancreatitis or hyperadrenocorticism. Conversely, Staffordshire Bull Terriers (SBT), Shih-tzus and German Shepherd Dogs (GSDs) were less likely to develop DM.

For the survival of dogs with DM, factors associated with decreased survival included dogs > 10 years old at diagnosis, Cocker Spaniels, those with very high blood glucose readings at diagnosis with DM, or those who had previously been on glucocorticoid (steroid) medication. Factors associated with increased survival included dogs that were neutered, Border Collies and dogs starting insulin treatment.

## Background

Diabetes mellitus (DM) is a relatively common endocrinopathy of dogs, with an estimated prevalence of approximately 0.32–0.36% [1–3]. Clinical DM in dogs is characterised by the loss of pancreatic islet cells resulting in insulin deficiency and persistent hyperglycaemia, resulting in clinical signs including polyuria, polydipsia, polyphagia and weight loss [4, 5]. Both genetic and environmental factors are implicated in the development of this disease [6]. Although the exact pathogenesis leading to islet cell loss is often unclear [6], and is likely to be heterogeneous, there are thought to be similarities between some cases of DM in dogs and type 1 diabetes mellitus (T1DM) in humans [7–9]. The incidence of T1DM has been increasing worldwide [10], and the speed of this rise suggests it is not solely related to genetic factors. The prevalence of DM in dogs is reported to be increasing, up by 79.7% since 2006 in the US [11, 12], and highlights a need for a greater understanding of the current frequency and risk factors in the development of the disease.

Factors reported to be associated with the development of DM in dogs include genetics, age, sex, neutering status, obesity, drug therapy, infection and concurrent disease [9, 13]. Juvenile-onset diabetes before 1 year of age in dogs is uncommon [1], and is more likely to have a familial element [14]. DM is more commonly diagnosed in middle-aged to older dogs [8, 11, 15], typically in dogs over 5 years of age. Although reported breed predispositions vary between countries, certain breeds appear predisposed including Samoyeds [7, 11, 16, 17], miniature schnauzers [7, 11, 17], Cairn terriers [11, 13, 15, 17–19] and Yorkshire terriers [2, 14]. Conversely German Shepherd Dogs (GSD), Golden retrievers [2, 17] and Boxers appear at a reduced risk [3, 13, 15, 16]. There has been inconclusive evidence for sex and neuter status as risk factors, likely related to the varying neutering practices internationally. Where it is not common practice to neuter female dogs, dioestrus-associated diabetes is more common. This is related to the antagonising effects on insulin from progesterone, as well as growth hormone released from the canine mammary glands under the influence of progesterone [20]. Some studies show females at increased risk compared to males [11, 15, 16, 21], and neutered males at increased risk compared to entire males [2, 3, 11]. However it is unclear whether sex alone is associated with DM [1–3, 14, 19, 22], and some studies also failed to identify association with neutering, though may be limited by their small sample sizes [19, 22].

Obesity has been associated with both human type 2 diabetes mellitus (T2DM), and feline diabetes [9], but there is limited evidence about the role of obesity in the development of DM in dogs [6, 23]. Obesity-induced insulin resistance has been documented in dogs, but it appears that dogs are resistant to developing type 2 diabetes [24]. A number of studies have identified being overweight/obese as risk factors for DM in dogs [2, 25, 26]. Conversely one small study reported an association with underweight dogs, but because body condition score (BCS) was assessed by the veterinarian at the time of diagnosis, this may reflect consequential weight loss associated with the DM rather than as a true predictive risk factor [19].

Diabetes mellitus in dogs has been associated with co-morbidities such as hyperadrenocorticism, urinary tract infections (UTI), dermatitis, otitis, pancreatitis and hypothyroidism [2, 3, 14, 17, 27]. Hyperadrenocorticism is the most commonly associated endocrinopathy with DM [17, 28, 29], and has been identified as a risk factor [2, 16], which is most likely related to the cortisol antagonism of insulin. Immune-mediated insulitis and exocrine pancreatic disease are also thought to play a role in the pathogenesis of DM [1, 30, 31] and, although the exact relationship between the two diseases is not entirely clear [32], pancreatitis is commonly found concurrently with DM [2, 3, 17, 33, 34]. One study found pancreatitis was associated with increased risk of DM, and with decreased survival [2].

The few studies that have reported on the survival of dogs with DM have provided little agreement on the median survival time (MST) [2, 16, 34, 35]. A study of insured dogs in Sweden reported a MST of 57 days from the date of the first insurance claim across all cases, increasing to 2 years for dogs surviving at least 1 day [16]. Comparatively, a study of English primary-care practices and a study from a referral hospital in Italy reported MST as 17.3 months [2] and 32 months [34] respectively. A study surveying veterinarians reported that 1 in 10 dogs were euthanised at diagnosis and another 1 in 10 euthanised within 1 year, most commonly due to concurrent conditions, cost considerations and age [35]. Little is known about the risk factors for survival in dogs with DM, particularly in a primary care setting. Pancreatitis and old age have been associated with a higher hazard of death, whereas neutered and insured dogs had lower hazard [2]. Another study found no association with age, nor with a previous diagnosis of pancreatitis, but breed affected survival time [16]. However these studies have been limited by relatively short follow up time [2, 16], or being restricted to referral populations [34], and none have investigated the prognostic impact of the initial management of the condition on subsequent survival.

The aim of this study was to estimate prevalence and incidence of DM in a large population of dogs under primary veterinary care in the UK, and to investigate risk factors for DM and the survival of DM cases. Secondary aims were to describe the current diagnostic processes and early DM management as well as exploring associations between these and survival. A greater understanding of the risk factors involved in the development of DM, and prognostic indicators for survival, can help inform genetic studies and allow population stratification for clinical trials as well as aiding primary care clinicians in identifying individuals at risk and in providing owners with prognostic information.

## Results

The study population consisted of 480,469 dogs aged ≥3 years on 01/01/2016 under veterinary care at 430 primary-care UK-wide VetCompass practices during the study period. The median age was 6.7 years (range: 3.0–20.2 years), 48.2% (231,524) were female, and 56.4% (271,068) were neutered. Within this population, 409 new (incident) and 863 pre-existing DM cases (total 1272) were identified in 2016, giving an apparent annual prevalence of 0.26% (95% confidence interval (CI): 0.25–0.28%), and an annual incidence risk of 0.09% (95%CI: 0.08–0.09%) in dogs aged ≥3 years.

### Descriptive statistics

Of the 409 incident DM cases in 2016, 48.9% (200) were female, and 70.7% (289) were neutered, 77.5% (317) were classified purebred and 32.5% (133) were insured. The median age at diagnosis was 10.0 years (range: 3.2–18.0 years). For those dogs where diagnostic test information was recorded electronically (392), nearly all diagnoses included blood testing (96.9%, 380/392), and the majority had a combination of blood testing and urinalysis (76.0%, 298) (Table 1). Dogs that were ketotic at diagnosis (32.8%, 134), and dogs with cataracts present within 3 months after diagnosis (32.8%, 134) both accounted for approximately a third of cases each. Recorded blood glucose (BG) levels at diagnosis ranged between 12.2–51.7 mmol/L, median 28.1 mmol/L. Most dogs (90.1%, 362) were started on insulin treatment, most commonly twice daily injections (68.3%, 224). There was no information on insulin treatment for 1.7% [7] of dogs. Of those dogs not receiving insulin treatment (9.8%, 40), only 17.5% (7/40) survived > 7 days, and of these dogs only 1 received another drug (acarbose). There were 16.1% (66) of dogs hospitalised at diagnosis, and only 5.9% [24] were referred for advanced management.

**Table 1:** Diagnostics and management techniques for dogs diagnosed with diabetes mellitus

	Number of cases (%) (***n*** = 409)
**Diagnostic procedures (multiple tests allowed per dog)**	
Urinalysis	310 (75.8%)
Blood Glucose (BG)	356 (87.0%)
Fructosamine	169 (41.3%)
Blood (unspecified)	330 (80.7%)
Other^a^	36 (8.8%)
Diagnostic tests not recorded	17 (4.2%)
**Blood glucose level at diagnosis**	
Median (range) mmol/L	28.1 (12.2–51.7)
< 20 mmol/L	34 (8.3%)
20 to < 30 mmol/L	108 (26.4%)
30 to < 40 mmol/L	83 (20.3%)
> 40 mmol/L	23 (5.6%)
Level unrecorded	161 (39.4%)
**Ketotic at diagnosis**	
Ketotic	134 (32.8%)
Not ketotic	263 (43.8%)
No record of assessing ketones	96 (23.5%)
**Insulin treatment**	
Dog started on insulin	362 (88.5%)
Dog not started on insulin^b^	40 (9.8%)
Insulin treatment unknown	7 (1.7%)
**Insulin regime**	
Once a day	67 (16.4%)
Twice a day	224 (54.8%)
> 2x daily injections	24 (5.9%)
Constant Rate Infusion	13 (3.2%)
Unable to determine initial insulin regime	81 (19.8%)
**Cataracts present**	
Cataracts diagnosed	134 (32.8%)
Not recorded	275 (67.2%)
**Hospitalised at diagnosis**	
Dog hospitalised	66 (16.1%)
No evidence of hospitalisation	343 (83.9%)
**Number of days hospitalised**	
Median (range)	0 (0–12)
0 days	343 (83.9%)
1–3 days	49 (12.0%)
4+ days	17 (4.2%)
**Referred for advanced management**	
Dog referred	24 (5.9%)
Not referred	385 (94.1%)
**Management methods first 3 months (multiple methods allowed per dog)**	***n*** **= 361**
Home blood glucose measurements	45 (12.5%)
Blood glucose curve practice	234 (64.8%)
Fructosamine	135 (37.4%)
Spot blood glucose	247 (68.4%)
Bloods (unspecified)	110 (30.5%)
Urinalysis	160 (44.3%)
Recommended diet change	149 (41.3%)
Other^c^	128 (35.5%)

Diagnostics and management for dogs aged 3 years and older diagnosed with diabetes mellitus in UK primary-care practices in 2016.

^a^ The majority of “other” tests (91.7%, 33 dogs) were imaging

^b^ Dogs not started on insulin included those where the owner opted for euthanasia, or specifically declined insulin treatment

^c^ “Other” management included 92.2% (118) fluid therapy, 10.2% [13] imaging

Where monitoring of DM in the first 3 months was recorded (88.3%, 361), 90% primarily involved blood testing (324/361), which consisted of haematology, biochemistry, BG measurements, and/or fructosamine analyses. The most common monitoring approaches were home or practice BG curves (70.6%, 255) and/or spot BG (68.4%, 247). Only 15 (4.2%) of the dogs were managed with spot BG alone and no other tests. Almost half of dogs (44.3%, 160) had urinalysis as part of their monitoring, and a similar percentage (41.3%, 149) had a diet change recommended. “Other” management techniques were used in 35.5% (128) of cases, and in most cases, this was fluid therapy.

### Case-control study

The results of univariable logistic analysis are described in Table 2. There were strong associations with the following variables: age at diagnosis, neutering status, sex combined with neutering status, bodyweight, breed, obesity, prior treatment with glucocorticoids, a concurrent diagnosis of hyperadrenocorticism or pancreatitis, insurance status and veterinary group. Purebred status was associated at *p* < 0.2, and sex alone was not associated with diagnosis of DM.

**Table 2:** Descriptive and univariable logistic regression results

Variable	Case (%) ***n*** = 409	Control (%) ***n*** = 818	Odds Ratio	95% CI^a^	Category ***P***-value	Variable ***P***-value
**Age** Median (range)	10.00 (3.16–18.00)	7.12 (3.07–17.25)				< 0.001
**Age at diagnosis**						
3 to < 8 years	77 (18.8%)	464 (56.7%)	Base			< 0.001
8 to < 10 years	124 (30.3%)	153 (18.7%)	4.88	3.48–6.85	< 0.001	
10 to < 13 years	165 (40.3%)	140 (17.1%)	7.10	5.11–9.88	< 0.001	
> 13 years	43 (10.5%)	61 (7.5%)	4.25	2.68–6.72	< 0.001	
**Sex**		***n*** **= 817**				0.732
Female	200 (48.9%)	408 (49.9%)	Base			
Male	209 (51.1%)	409 (50.1%)	1.04	0.82–1.32	0.732	
**Neuter status**		**n = 817**				0.001
Entire	120 (29.3%)	322 (39.4%)	Base			
Neutered	289 (70.7%)	495 (60.6%)	1.57	1.21–2.02	0.001	
**Sex-neuter**		**n = 817**				< 0.001
Male-entire	49 (12.0%)	173 (21.2%)	Base			
Male-neutered	160 (39.1%)	236 (28.9%)	2.39	1.64–3.48	< 0.001	
Female-entire	78 (19.1%)	149 (18.2%)	1.85	1.22–2.81	0.004	
Female-neutered	122 (29.8%)	259 (31.7%)	1.66	1.13–2.44	0.009	
**Weight: PUREBRED ONLY**	***n*** **= 253**	***n*** **= 485**				< 0.001
Below breed mean	92 (36.4%)	246 (50.7%)	Base			
At or above breed mean	161 (63.6%)	239 (49.3%)	1.80	1.32–2.46	< 0.001	
**Purebred status**						0.051
Crossbred	92 (22.5%)	226 (27.7%)	Base			
Purebred	317 (77.5%)	591 (72.3%)	1.15	1.00–1.32	0.051	
**Breed ≥ 10 dogs and/or ≥ 5 case dogs**						< 0.001
Crossbred	88 (21.5%)	204 (24.9%)	Base			
Tibetan Terrier	9 (2.2%)	2 (0.2%)	10.43	2.21–49.27	0.003	
Border Terrier	21 (5.1%)	10 (1.2%)	4.87	2.20–10.76	< 0.001	
Cairn Terrier	5 (1.2%)	3 (0.4%)	3.86	0.90–16.52	0.068	
Miniature Schnauzer	10 (2.4%)	6 (0.7%)	3.86	1.36–10.96	0.011	
West Highland White Terrier	53 (13.0%)	32 (3.9%)	3.84	2.32–6.36	< 0.001	
Yorkshire Terrier	30 (7.3%)	23 (2.8%)	3.02	1.66–5.50	< 0.001	
Bichon Frise	11 (2.7%)	15 (1.8%)	1.70	0.75–3.85	0.203	
Cavalier King Charles Spaniel	16 (3.9%)	22 (2.7%)	1.69	0.84–3.36	0.138	
Jack Russell Terrier	32 (7.8%)	51 (6.2%)	1.45	0.88–2.42	0.148	
Border Collie	13 (3.2%)	23 (2.8%)	1.31	0.63–2.70	0.465	
Cocker Spaniel	13 (3.2%)	26 (3.2%)	1.16	0.57–2.36	0.684	
Purebred (other)	71 (17.4%)	269 (32.9%)	0.90	0.61–1.33	0.597	
Labrador Retriever	25 (6.1%)	76 (9.3%)	0.76	0.45–1.28	0.304	
Lhasa Apso	4 (1.0%)	13 (1.6%)	0.71	0.23–2.25	0.564	
Staffordshire Bull Terrier	12 (2.9%)	56 (6.9%)	0.50	0.25–0.97	0.041	
Springer Spaniel – unspecified	2 (0.5%)	12 (1.5%)	0.39	0.08–1.76	0.219	
Golden Retriever	1 (0.2%)	9 (1.1%)	0.26	0.03–2.06	0.201	
Boxer	1 (0.2%)	11 (1.35)	0.21	0.03–1.66	0.139	
Shih-Tzu	2 (0.5%)	24 (2.9%)	0.19	0.04–0.84	0.028	
Chihuahua	1 (0.2%)	14 (1.7%)	0.17	0.02–1.28	0.085	
German Shepherd Dog	1 (0.2%)	14 (1.7%)	0.17	0.02–1.28	0.085	
English Springer Spaniel	0 (0.0%)	10 (1.2%)	.	.	.	
Pug	0 (0.0%)	10 (1.2%)	.	.	.	
**Co-morbidities/medication**						
Glucocorticoids: Yes	34 (8.3%)	28 (3.4%)	2.56	1.53–4.28	< 0.001	< 0.001
No	375 (91.7%)	790 (96.6%)	Base			
Obesity: Yes	67 (16.4%)	79 (9.7%)	1.83	1.29–2.60	0.001	0.001
No	342 (83.6%)	739 (90.3%)	Base			
Hyperadrenocorticism: Yes	26 (6.4%)	2 (0.2%)	27.7	6.54–117.29	< 0.001	< 0.001
No	383 (93.6%)	816 (99.8%)	Base			
Pancreatitis: Yes	46 (11.3%)	1 (0.1%)	103.53	14.22–753.62	< 0.001	< 0.001
No	363 (88.8%)	817 (99.9%)	Base			
**Insurance status**		**n = 817**				< 0.001
Insured	133 (32.5%)	113 (13.8%)	3.00	2.25–4.00	< 0.001	
Not Insured	276 (67.5%)	704 (86.2%)	Base			
**Veterinary group**						< 0.001
A	3 (0.7%)	3 (0.4%)	0.82	0.16–4.10	0.805	
B	147 (35.9%)	244 (29.9%)	0.49	0.37–0.66	< 0.001	
C	55 (13.5%)	40 (4.9%)	1.12	0.71–1.77	0.621	
D	201 (49.1%)	164 (20.1%)	Base			
E	3 (0.7%)	366 (44.8%)	0.01	0.002–0.02	< 0.001	

Descriptive statistics and univariable logistic regression for variables associated with diabetes mellitus in dogs aged 3 years and older attending UK primary-care practices in 2016.

^a^ Confidence Interval

The final multivariable model (Table 3) included eight variables, and appeared to explain the data well (Hosmer-Lemeshow *p* = 0.999). Clustering at clinic level was not significant when clinic ID was added as a random effect (*p* = 0.497). Veterinary group confounded associations with age at diagnosis, sex combined with neutering, concurrent conditions, and breed, and was therefore included as a fixed effect. After adjusting for the other variables in the model, an increased odds for DM diagnosis was seen with age > 8 years old (OR peaking at 10 to < 13 years old), and female entire dogs (OR: 3.03, 95% CI 1.69–5.44, *p* < 0.001) and male neutered dogs (OR: 1.99, 95% CI 1.18–3.34, *p* = 0.010) compared to male entire dogs. With male entire dogs as a baseline category in the sex-neuter variable there was no significant difference between the ORs for female entire dogs (OR: 3.03, 95% CI 1.69–5.44) and female neutered dogs (OR: 1.36, 95% CI 0.80–2.31). Border Terriers (OR: 3.37, 95% CI 1.04–10.98, *p* = 0.043) and West Highland White Terriers (WHWT) (OR: 2.88, 95% CI 1.49–5.56, *p* = 0.002) compared to crossbreeds were also associated with increased odds for DM, as were those documented obese (OR: 2.71, 95% CI 1.63–4.52, *p* < 0.001), or had a concurrent diagnosis of pancreatitis (OR: 1085.19, 95% CI 36.36–32,390.61, *p* < 0.001) or hyperadrenocorticism (OR: 11.28, 95% CI 2.41–52.73, *p* = 0.002). Compared to crossbreds, breeds with reduced odds of DM included Staffordshire Bull Terriers (SBT) (OR: 0.42, 95% CI 0.18–0.98, *p* = 0.046), Shih-tzu (OR: 0.20, 95% CI 0.04–0.96, *p* = 0.045) and German Shepherd Dogs (GSD) (OR: 0.08, 95% CI 0.01–0.74, *p* = 0.025).

**Table 3:** Multivariable logistic regression results

Variable	Odds Ratio	95% CI^a^	Category ***P***-value	Variable ***P***-value
**Age at diagnosis**				< 0.001
3 to < 8 years	Base			
8 to < 10 years	4.04	2.63–6.23	< 0.001	
10 to < 13 years	7.18	4.60–11.20	< 0.001	
> 13 years	3.55	1.97–6.40	< 0.001	
**Sex-neuter**				< 0.001
Male-entire	Base			
Male-neutered	1.99	1.18–3.34	0.010	
Female-entire	3.03	1.69–5.44	< 0.001	
Female-neutered	1.36	0.80–2.31	0.250	
**Breed ≥ 10 dogs and/or ≥ 5 case dogs**				< 0.001
Tibetan Terrier	8.48	0.94–76.76	0.057	
Miniature Schnauzer	4.09	0.90–18.55	0.068	
Border Terrier	3.37	1.04–10.98	0.043	
West Highland White Terrier	2.88	1.49–5.56	0.002	
Cavalier King Charles Spaniel	2.43	0.95–6.20	0.064	
Cairn Terrier	2.25	0.38–13.38	0.372	
Yorkshire Terrier	2.09	0.93–4.69	0.073	
Border Collie	1.57	0.58–4.27	0.378	
Bichon Frise	1.47	0.40–5.41	0.559	
Springer Spaniel – unspecified	1.20	0.15–9.70	0.864	
Cocker Spaniel	1.13	0.48–2.66	0.782	
Jack Russell Terrier	1.04	0.54–1.98	0.913	
Crossbred	Base			
Purebred (other)	0.87	0.52–1.44	0.589	
Labrador Retriever	0.83	0.43–1.57	0.559	
Lhasa Apso	0.65	0.11–3.73	0.624	
Staffordshire Bull Terrier	0.42	0.18–0.98	0.046	
Boxer	0.21	0.02–2.18	0.193	
Chihuahua	0.20	0.02–2.09	0.177	
Shih-tzu	0.20	0.04–0.96	0.045	
Golden Retriever	0.17	0.02–1.75	0.137	
German Shepherd Dog	0.08	0.01–0.74	0.025	
English Springer Spaniel	.	.	.	
Pug	.	.	.	
**Concurrent conditions/medication:**				
Glucocorticoid treatment: Yes	2.19	1.02–4.70	0.044	0.044
No	Base			
Obesity mentioned 1 yr prior to diagnosis: Yes	2.71	1.63–4.52	< 0.001	< 0.001
No	Base			
Hyperadrenocorticism +/− 3 months diagnosis: Yes	11.28	2.41–52.73	0.002	0.002
No	Base			
Pancreatitis +/− 3 months diagnosis: Yes	1085.19	36.36–32,390.61	< 0.001	< 0.001
No	Base			
**Veterinary group**				< 0.001
A	1.06	0.16–7.11	0.955	
B	0.58	0.41–0.83	0.003	
C	1.14	0.66–1.99	0.631	
D	Base			
E	0.003	0.0004–0.02	< 0.001	

Multivariable logistic regression for risk factors associated with diabetes mellitus in dogs 3 years and older attending UK primary-care practices in 2016, *n* = 1205.

^a^ Confidence Interval

### Survival analysis

There were 252 (61.6%) deaths from all-cause mortality prior to February 2020; of these, 147 (58.3%) were directly attributable to DM and 233 (92.5%) were euthanised. More than one reason was often given for a euthanasia decision, and the most frequent reason cited was worsening of DM clinical signs, cited in 58.4% (136/233) of euthanasia decisions (Table 4). This was closely followed by contributory conditions which were cited in 57.1% (133/233) of euthanasia decisions. Contributory conditions most frequently included ocular disorders, contributing to 14.2% (33/233) of euthanasia decisions, followed by pancreatitis, neoplasia and non-specific poor quality of life, each contributing to 7.3% (17/233) of all euthanasia decisions.

**Table 4:** Euthanasia reasons for dogs diagnosed with diabetes mellitus

Reasons for euthanasia (multiple allowed):		Number of cases (%) ***n*** = 233
Condition worsening		136 (58.4%)
Owner not coping with condition		15 (6.4%)
Financial		25 (10.7%)
Contributary conditions		133 (57.1%)
No reason given		44 (18.9%)
**Contributory conditions listed as (multiple allowed)**		
Ocular disorder	all	33 (14.2%)
	cataracts/ blindness	26 (11.2%)
Pancreatitis		17 (7.3%)
Neoplasia		17 (7.3%)
Poor quality of life/deterioration – unspecific		17 (7.3%)
Kidney disease		13 (5.6%)
Neurological condition	all	12 (5.2%)
	seizures	7 (3.0%)
Liver disease		10 (4.3%)
Diabetic ketoacidosis		8 (3.4%)
Hyperadrenocorticism		7 (3.0%)
Blood disorder		5 (2.1%)
Temperament of the dog		2 (0.9%)

Euthanasia reasons for dogs aged 3 years and older diagnosed with diabetes mellitus attending UK primary-care practices in 2016, *n* = 233.

Median survival time from diagnosis for all dogs was 15.6 months (95% CI: 10.4–20.0), and for those surviving at least 7 days post DM diagnosis, MST was 20.2 months (95% CI: 16.6–24.7).

The results from univariable cox regression are described in Table 5. There were strong associations (*p* ≤ 0.001) with survival for the following variables: age, neutering status, insulin treatment, and monitoring methods that included BG curves at the practice, spot BG, fructosamine measurements or a recommended diet change. Other variables associated at p < 0.2 included: sex, combined sex and neuter status, insurance, breed, being ketotic at diagnosis, prior glucocorticoid treatment, obesity, BG level at diagnosis, and monitoring methods including bloods (unspecified) and urinalysis.

**Table 5:** Univariable cox regression results

Variable	Number (%) ***n*** = 409	Hazard Ratio	95% CI^**a**^	Category ***P***-value	Variable ***P***-value
**Age at diagnosis** Median (range)	10.00 (3.16–18.00)				< 0.001
**Age at diagnosis**					
3 to < 8 years	77 (18.8%)	Base			< 0.001
8 to < 10 years	124 (30.3%)	1.05	0.70–1.59	0.805	
10 to < 13 years	165 (40.3%)	2.25	1.54–3.29	< 0.001	
> 13 years	43 (10.5%)	2.69	1.64–4.43	< 0.001	
**Sex**					0.277
Female	200 (48.9%)	Base			
Male	209 (51.1%)	0.87	0.68–1.12	0.277	
**Neuter status**					0.001
Entire	120 (29.3%)	Base			
Neutered	289 (70.7%)	0.62	0.47–0.81	0.001	
**Sex-neuter**					0.072
Male-entire	49 (12.0%)	Base			
Male-neutered	160 (39.1%)	0.63	0.42–0.95	0.029	
Female-entire	78 (19.1%)	0.92	0.58–1.47	0.738	
Female-neutered	122 (29.8%)	0.74	0.48–1.12	0.155	
**Purebred status**					
Crossbred	92 (22.5%)	Base			0.895
Purebred	317 (77.5%)	0.99	0.86–1.15	0.895	
**Weight: PUREBRED ONLY**	**n = 253**				
Below breed mean	92 (36.4%)	Base			0.459
At or above breed mean	161 (63.6%)	0.88	0.63–1.23	0.459	
**Breed (≥5 dogs per breed)**					
Crossbred	88 (21.5%)	Base			0.029
Staffordshire Bull Terrier	9 (2.2%)	2.16	1.02–4.55	0.043	
Cocker Spaniel	21 (5.1%)	1.61	0.85–3.07	0.147	
Border Terrier	5 (1.2%)	1.40	0.80–2.43	0.239	
Jack Russell Terrier	10 (2.4%)	1.20	0.72–1.99	0.487	
Purebred (other)	53 (13.0%)	1.11	0.73–1.67	0.631	
Labrador Retriever	30 (7.3%)	1.16	0.67–1.99	0.599	
Tibetan Terrier	11 (2.7%)	0.81	0.33–2.02	0.653	
West Highland White Terrier	16 (3.9%)	1.07	0.70–1.64	0.753	
Cavalier King Charles Spaniel	32 (7.8%)	0.67	0.32–1.41	0.291	
Miniature Schnauzer	13 (3.2%)	0.82	0.35–1.89	0.636	
Yorkshire Terrier	13 (3.2%)	0.63	0.36–1.09	0.097	
Cairn Terrier	25 (6.1%)	0.46	0.11–1.89	0.281	
Border Collie	71 (17.4%)	0.47	0.21–1.03	0.060	
Bichon Frise	12 (2.9%)	0.43	0.17–1.07	0.069	
**Co-morbidities/medication:**					
Glucocorticoids Yes	34 (8.3%)	1.70	1.14–2.52		0.014
No	375 (91.7%)	Base		0.014	
Obesity Yes	67 (16.4%)	0.67	0.47–0.96		0.023
No	342 (83.6%)	Base		0.023	
Hyperadrenocorticism Yes	26 (6.4%)	1.33	0.82–2.15		0.263
No	383 (93.6%)	Base		0.263	
Pancreatitis Yes	46 (11.3%)	1.11	0.76–1.61		0.593
No	363 (88.8%)	Base		0.593	
**Insurance status**					
Insured	133 (32.5%)	0.86	0.67–1.12		0.267
Not Insured	276 (67.5%)	Base		0.267	
**Veterinary group**					0.8437
A	3 (0.7%)	1.08	0.15–7.71	0.941	
B	147 (35.9%)	0.89	0.67–1.18	0.403	
C	55 (13.5%)	1.12	0.77–1.63	0.549	
D	201 (49.1%)	Base			
E	3 (0.7%)	1.09	0.27–4.41	0.903	
**BG level at diagnosis**					0.003
Median (range) mmol/L	28.1 (12.2–51.7)				
< 20 mmol/L	34 (8.3%)	Base			
20 to < 30 mmol/L	108 (26.4%)	0.93	0.55–1.57	0.774	
30 to < 40 mmol/L	83 (20.3%)	1.53	0.90–2.61	0.118	
> 40 mmol/L	23 (5.6%)	2.48	1.29–4.78	0.007	
No evidence/record of BG level	161 (39.4%)	1.37	0.83–2.27	0.215	
**Ketotic at diagnosis**					0.095
Ketotic	134 (32.8%)	1.15	0.86–1.54	0.332	
Not ketotic	179 (43.8%)	Base			
No record of assessing ketones	96 (23.5%)	1.42	1.04–1.95	0.028	
**Cataracts diagnosed within 3 months of diagnosis**					0.500
Cataracts diagnosed	134 (32.8%)	0.91	0.70–1.19	0.502	
No mention of cataracts	275 (67.2%)	Base			
**Hospitalised at diagnosis**					0.866
Dog hospitalised	66 (16.1%)	0.97	0.69–1.36	0.867	
No evidence of hospitalisation	343 (83.9%)	Base			
**Referred for advanced management**					0.991
Referred	24 (6.0%)	1.00	0.57–1.75	0.991	
Not referred	378 (94.0%)	Base			
**Insulin treatment**					< 0.001
Treated with insulin	362 (88.5%)	0.08	0.05–0.12	< 0.001	
Not treated	40 (9.8%)	Base			
Insulin treatment unknown	7 (1.7%)	.	.	.	
**Initial insulin regime**	***n*** **= 328**				0.883
Once daily injections	67 (20.4%)	0.91	0.62–1.32	0.612	
Twice daily injections	224 (68.3%)	Base			
> 2x daily injections	24 (7.3%)	1.17	0.68–2.03	0.571	
Constant Rate Infusion	13 (4.0%)	0.96	0.45–2.06	0.921	
**MANAGEMENT (multiple methods per dog)**					
Home blood glucose measurements: Yes	45 (11.0%)	1.06	0.74–1.52	0.733	0.735
No	338 (89.0%)	Base			
Blood glucose curve practice: Yes	234 (57.2%)	0.51	0.39–0.65	< 0.001	< 0.001
No	175 (42.8%)	Base			
Fructosamine: Yes	135 (33.0%)	0.65	0.50–0.85	0.001	0.001
No	274 (67.0%)	Base			
Spot blood glucose: Yes	247 (60.4%)	0.66	0.51–0.85	0.001	0.001
No	162 (39.6%)	Base			
Bloods (unspecified): Yes	110 (26.9%)	0.82	0.62–1.09	0.171	0.164
No	299 (73.1%)	Base			
Urinalysis: Yes	160 (39.1%)	0.79	0.61–1.01	0.063	0.061
No	249 (60.9%)	Base			
Recommended diet change: Yes	149 (36.4%)	0.53	0.41–0.70	< 0.001	< 0.001
No	260 (63.6%)	Base			
Other (Fluid therapy/imaging): Yes	108 (26.4%)	1.07	0.81–1.41	0.632	0.632
No	301 (73.6%)	Base			

Univariable cox regression survival analysis for dogs 3 years and older, diagnosed with diabetes mellitus at UK primary-care practices in 2016.

^**a**^ Confidence Interval

The final multivariable model showed survival to be associated (*p* < 0.05) with age, neutering status, insulin treatment, prior glucocorticoid treatment, and BG level at diagnosis (Table 6). Dogs within the following categories showed increased hazard of death following diagnosis of DM: both age categories ≥10 years of age compared to those aged 3 - < 8 years (10 - < 13 years HR: 2.12, 95% CI 1.42–3.18; > 13 years HR: 2.02, 95% CI 1.17–3.49), Cocker Spaniels (HR: 2.06, 95% CI 1.06–4.01, *p* = 0.034) compared to crossbreeds, a BG level at diagnosis > 40 mmol/L (HR: 2.75, 95% CI 1.35–5.57, *p* = 0.005) compared to < 20 mmol/L at diagnosis, or previous glucocorticoid treatment (HR: 1.83, 95% CI 1.20–2.80, *p* = 0.005). Factors associated with reduced hazard of death were neutered dogs (HR: 0.56, 95% CI 0.42–0.78, *p* < 0.001), Border Collies (HR: 0.39, 95% CI 0.17–0.87, *p* = 0.022) and dogs starting insulin treatment (HR: 0.08 95% CI 0.05–0.12, *p* < 0.001). Initiation of insulin treatment was associated with a variety of management methods in the first 3 months. To avoid collinearity, only insulin treatment was included in the final model. However the following management methods were all associated with increased survival when substituting them for insulin treatment and after adjusting for the other variables in the final model: BG curves at the practice (HR: 0.52, 95% CI 0.40–0.68, p < 0.001), recommended diet change (HR: 0.61, 95% CI 0.46–0.81, *p* = 0.001), fructosamine measurements (HR: 0.63, 95% CI 0.48–0.84, p = 0.002) and spot BG measurements (HR: 0.67, 95% CI 0.51–0.89, *p* = 0.004).

**Table 6:** Multivariable cox regression results

Variable	Hazard Ratio (***n*** = 409)	95% CI^**a**^	Category P-value	Variable ***P***-value
**Age at diagnosis**				< 0.001
3 to < 8 years	Base			
8 to < 10 years	0.98	0.64–1.50	0.935	
10 to < 13 years	2.12	1.42–3.18	< 0.001	
> 13 years	2.02	1.17–3.49	0.011	
**Neuter status**				< 0.001
Entire	Base			
Neutered	0.56	0.42–0.78	< 0.001	
**Breed ≥ 5 dogs**				0.050
Cocker Spaniel	2.06	1.06–4.01	0.034	
Staffordshire Bull Terrier	1.85	0.86–3.97	0.116	
Border Terrier	1.19	0.68–2.10	0.543	
Labrador Retriever	1.08	0.62–1.89	0.786	
Purebred (other)	1.00	0.63–1.57	0.989	
Crossbred	Base			
Miniature Schnauzer	0.96	0.41–2.25	0.925	
Jack Russell Terrier	0.88	0.51–1.52	0.655	
Cavalier King Charles Spaniel	0.86	0.40–1.84	0.690	
Tibetan Terrier	0.72	0.28–1.84	0.489	
Yorkshire Terrier	0.69	0.39–1.21	0.198	
West Highland White Terrier	0.69	0.44–1.08	0.103	
Cairn Terrier	0.56	0.13–2.37	0.432	
Bichon Frise	0.46	0.18–1.16	0.101	
Border Collie	0.39	0.17–0.87	0.022	
**BG level at diagnosis**				0.004
< 20 mmol/L	Base			
20 to < 30 mmol/L	0.96	0.55–1.70	0.913	
30 to < 40 mmol/L	1.52	0.88–2.65	0.137	
> 40 mmol/L	2.75	1.35–5.57	0.005	
No evidence/record of BG test	1.42	0.84–2.41	0.187	
**Insulin treatment**				< 0.001
Insulin treatment started	0.08	0.05–0.12	< 0.001	
No insulin treatment	Base			
Insulin treatment unknown	.	.	.	
**Glucocorticoid treatment 6 wks prior to diagnosis**				0.008
Yes	1.83	1.20–2.80	0.005	
No	Base			
**Veterinary group**				0.087
A	2.12	0.26–17.38	0.488	
B	0.75	0.56–1.02	0.063	
C	1.35	0.91–2.02	0.137	
D	Base			
E	1.64	0.39–6.99	0.501	

Multivariable cox regression survival analysis for dogs 3 years and older diagnosed with diabetes mellitus at UK primary-care practices in 2016.

^**a**^ Confidence Interval

The clinic ID frailty term in the final multivariable model was not statistically significant (*P* = 0.497). Veterinary group confounded both neutering and breed associations with survival, and was therefore included. Inspection of the Nelson-Aalen cumulative hazard plots and Schoenfeld residuals (*P* = 0.113) suggested the proportional hazards assumption was not violated.

## Discussion

This is the largest study to date exploring risk factors for DM in dogs in primary-care practices. It is the first to look at data from across the whole of the UK, and to explore associations between initial management methods for DM and survival. Dogs > 8 years of age, female entire dogs, male neutered dogs, WHWT and Border Terrier breeds in particular, dogs documented as obese, or having a concurrent diagnosis of hyperadrenocorticism or pancreatitis were all associated with an increased odds of DM diagnosis. Variables associated with an increased hazard of death after diagnosis included dogs that were ≥ 10 years of age at diagnosis, entire, previously on glucocorticoids, having had a BG level > 40 mmol/L at diagnosis, or dogs that did not start insulin treatment.

The prevalence of DM in the current study (0.26%) was calculated for dogs aged 3 years or above, and therefore may not be directly comparable to the higher prevalence values of 0.32–0.36% reported by other UK and Australian studies assessing the overall population dogs within a primary-care setting [1–3]. However the current study may offer a more accurate representation of the wider UK dog population, as previous studies were limited to only insured dogs [1], or only data from England [2]. Similarly, prevalence estimates of 0.64–1.33% reported by studies of hospital-based populations [11, 36] are likely to be biased due to the referred source population [37], and may not accurately reflect the wider dog population. In the current study, only 5.9% (24/409) of incident cases were referred for advanced management, highlighting the importance of studies from primary-care practice given that referral centres may be missing almost 95% of DM cases from the general dog population.

The annual incidence risk in dogs aged ≥3 years in the current study was estimated at 0.09% (95% CI: 0.08–0.09%). The study by Fall et al. (2007) on a Swedish insured dog population reported the cumulative proportion of dogs developing DM by 12 years of age as 1.2%, approximating to an annual incidence risk of 0.1% [38]. It is very difficult to compare these two studies due to the different source populations and methodologies for calculating annual incidence risk. Human T1DM had a reported average annual increase of 2.8% worldwide between 1990 and 1999 [10], and previous studies have suggested that the incidence of canine DM may also be increasing [11, 12].

Diagnostic testing information was available for 95.8% (392/409) DM cases in the current study. Of these dogs 32.8% (134) were ketotic at diagnosis which, having been assessed via urinary dipsticks, is likely to be an under-estimate because this test will not detect all types of ketone bodies [39]. This relatively high prevalence of ketosis at the time of diagnosis in primary care practice has not previously been reported. The same percentage of dogs (32.8%, 134) either presented with or developed cataracts within 3 months of DM diagnosis. The current study did not discriminate between diabetic and non-diabetic cataracts. However, some cases that developed cataracts may not have been documented in the clinical notes, suggesting that this figure could be an underestimate. Previous studies have reported 50% of diabetic dogs developing cataracts within 6 months [40, 41]. Although diabetic cataracts can be managed surgically, this is not always affordable for owners and ocular health was cited as a contributory reason for euthanasia in 14.2% (33/233) of all euthanasia decisions.

Age was strongly associated with the odds of diagnosis of DM. Consistent with other studies [2, 11], dogs over 8 years were at increased risk, with those aged 10 to < 13 years having over 7 times the odds of DM diagnosis compared to dogs aged 3 - < 8 years. No association was detected with sex, which is consistent with other studies [2, 14]. However, there was a strong association with the combined sex-neuter variable. Similar to previous findings, neutered males had almost twice the odds [2, 3, 11], and entire females had three times the odds of developing DM compared to entire males. Where there is a large entire female population, such as in Sweden, the increase in female cases of DM is thought to be a reflection of dioestrus diabetes [16]. However, despite the evidence between hormonal changes in dioestrus and the development of diabetes [20, 26, 42], overall the current study found no significant difference between entire and neutered females in developing DM. Instead, it suggests that entire males may be “protected”. Cross-sectional studies in humans have reported that men with lower testosterone levels have an increased T2DM risk [43]. In spontaneous mouse models of T1DM, such as the non-obese diabetic mouse, females are predisposed to diabetes, an effect thought to be mediated through testosterone-driven effects on the microbiome [44]. Although the pathogenesis of DM in dogs is heterogeneous, one mechanism by which male entire dogs may be relatively protected is via increased testosterone. However, this hypothesis may only be relevant to a proportion of diabetes cases, and was not investigated directly in the present study.

Breed associations were consistent with previous findings [3, 13, 15, 16], and add weight to the evidence that DM has a genetic component [7, 13]. To identify new genes and potential treatment targets in canine diabetes, understanding which breeds are genetically protected from DM is just as important as identifying those with a predisposition. In an aim to explore the effect of breed on both predisposition and protection in the current study, breeds with ≥10 dogs within cases and controls combined, and/or breeds with ≥5 DM cases were included as individual breeds within the ‘breed’ variable. Other breeds falling outside this definition were combined as ‘purebred other’. This enabled these individual breeds to be evaluated within the multivariable logistic analysis, and after adjustment for other variables and confounders on breed, aiming to provide a more accurate understanding of associations compared to most previous studies that use univariable analysis only. However, despite 1205 dogs being taken forward to multivariable analysis, this categorisation was still under-powered to evaluate breeds adequately where there were very few or no cases, despite the breed itself being relatively common. In this respect, this study was unable to identify all breeds at high risk of developing DM, and the findings focus on the breeds that were commonly represented in the UK during 2016.

The current study identified that WHWTs and Border Terriers were “at risk”, having approximately 3 times the odds of DM compared to crossbreds. Samoyeds are frequently over-represented in DM cases in other studies and although there was only one Samoyed present in this case control study, it was a DM case. Breeds with reduced odds of DM compared to crossbreds included GSD and Shih-Tzu, as well as SBT, a breed consistent with suggestive findings in other studies [2], but not previously significantly associated with lower DM risk. Interestingly the current study contained 10 English Springer Spaniels and 10 Pugs, but there were no cases documented for either of these breeds. Pugs have not previously been associated with a decreased odds of DM, whereas English Springer Spaniels have been associated with both a reduced odds of DM in the UK [13], and an increased odds of DM in Australia [3]. This suggests that different genetic subpopulations of English Springer Spaniels with different susceptibilities to DM may exist across these two geographical regions. This may be a useful area for further research into the genetics of DM in dogs.

Dogs documented as obese were associated with 2.7 times the odds of DM diagnosis (95% CI 1.63–4.52, *p* < 0.001). Despite no clear biological reason for obese dogs to be prone to DM due to insulin deficiency [24], this finding adds to evidence from other studies that obesity may be a risk factor for the disorder [2, 26], potentially by causing insulin resistance. The present study relied on subjective, unprompted recording of obesity by the veterinary professional, therefore under-reporting was likely, particularly with regards to controls, because cases had more detailed histories/examinations. Diet has also been associated with DM in dogs [26], and because obesity is often associated with poor dietary control and limited exercise [45], another explanation is that “obesity” may be acting as a proxy for other associated risk factors. It is clear that more research is required to establish the exact link between obesity in dogs and DM. Interestingly obesity was not associated with, nor confounded by, prior glucocorticoid treatment. The latter was associated with roughly double the odds of DM, which is likely to reflect insulin resistance caused by these drugs [46].

Concurrent hyperadrenocorticism and pancreatitis were both strongly associated with DM diagnosis, consistent with several other studies [2, 3, 17, 27]. The results should be interpreted with some caution because the numbers of controls with these conditions in this study were low, albeit consistent with the prevalence of these diseases (approx. 0.02–0.04%) in the wider UK dog population [47]. The temporality, and causation, between pancreatitis and DM is also difficult to determine [32], and interestingly only 13% (6/46) of the pancreatitis diagnoses in the current study clearly preceded DM. In total pancreatitis was documented in an unprompted way in 11.3% of cases, similar to the 11.5% reported in a previous UK primary-care study [2], but less than the 17.7% reported in DM cases in primary-care practice in Australia [3], and the 19% reported by an Italian referral study [34]. It is likely that pancreatitis is under-reported in the current study for both cases and controls due to non-specific clinical signs, and lack of definitive diagnostics being performed. Further research is required to fully understand the interplay between these two diseases.

Where recorded, the most common management methods for DM in the first 3 months were home or practice BG curves (70.6%, 255/361), followed by spot BG measurements (68.4%, 247), even though the latter is considered unreliable for monitoring [48]. Only 4.2% [15] of dogs were managed by spot BG alone, suggesting this method is primarily used as an augmentation to other management techniques.. Identification of management techniques and diagnostics currently employed by primary-care clinicians by this study provides a benchmark against which individuals and clinics can compare their own processes and practices.

Median survival time for all dogs with DM was 15.6 months (95% CI: 10.4–20.0 months). This estimate is likely to be negatively skewed by inclusion of dogs where DM management was not realistically attempted. To account for this, an MST was calculated for all dogs surviving at least 7 days post diagnosis, estimated at 20.2 months (95% CI: 16.6–24.7 months). The all dogs MST estimated in this study is consistent with the 17.3 months reported by an earlier VetCompass study [2], but differs substantially from the reported MST of 2 months from a population of insured Swedish dogs [16], and of 32 months for dogs presenting to a referral hospital in Italy [34]. This may reflect differences between countries, or that dogs presenting to a referral hospital are more likely to have motivated owners and access to gold standard level of care. Successful treatment of DM requires substantial owner commitment, and given that 92.5% of the dogs in the current study died due to an owner’s decision to euthanise, it is clear that MST is strongly influenced by a variety of owner-related factors such as their finances, lifestyle or perception of the condition.

Age at diagnosis, neuter status and breed have all previously been associated with DM survival [2, 16], and were also associated in the current study. Dogs aged ≥10 years and Cocker Spaniels had twice the hazard of death compared to dogs 3 to < 8 years and crossbreds respectively. Conversely, Border Collies and neutered animals had a lower hazard compared to crossbreds and entire animals. The differences in survival between breeds may reflect genetic differences involved in the pathogenesis of the disease. Interestingly previously studies have found a female predisposition to DM in Border Collies [13, 16], suggesting dioestrus diabetes may be more prevalent in this breed. In the current study 92.3% (12/13) of the Border Collie cases were females, with 6 of these were entire and 6 were neutered at the time of diagnosis. The increased survival found in this breed could therefore be partially related to survival associated with dioestrus diabetes cases, where remission may be possible following ovariohysterectomy. Whilst the clinical signs associated with canine DM, such as polyuria and polydipsia are relatively consistent, the underlying pathogenesis is relatively heterogeneous. More detailed and consistent clinical phenotyping at the time of diagnosis e.g. measurement of pancreatic inflammatory markers, would enable improved disease classification, allowing further insights to be gained into the relationship between underlying pathogenesis and survival.

Overall, neutered animals had a lower hazard of death once diagnosed (hazard ratio (HR): 0.56, 95% CI 0.42–0.78, *p* < 0.001) which is consistent with the findings of a previous UK primary-care study [2]. There is no clear reason whether this is biological or due to neutering acting as a proxy for some other measure such as the owner’s ability and willingness to treat DM.

Insurance status and concurrent pancreatitis have previously been associated with survival in primary-care practice [2]. Pancreatitis was cited as a cause of death in 7.3% (17/233) of euthanised cases, but in the current study did not identify an association between survival with insurance status, or the diagnosis of concurrent pancreatitis or hyperadrenocorticism at the time of DM diagnosis. A recent referral study similarly did not identify association with pancreatitis and survival [34], suggesting that pancreatitis, where recognised in the 3 months before or after diagnosis, may not significantly affect survival.

Other variables associated with survival in the current study included BG level at diagnosis, insulin treatment, and glucocorticoid treatment. Dogs with a BG reading > 40 mmol/L at diagnosis had a hazard ratio of 2.75 (95% CI: 1.35–5.57, *p* = 0.004) compared to dogs with a BG reading < 20 mmol/L. Higher BG levels at diagnosis may reflect “sicker” dogs or those in diabetic ketoacidosis, yet neither hospitalisation, or being ketotic at diagnosis, (a proxy measure for diabetic ketoacidosis) were associated with BG levels or survival. A referral study of incident cases reported that BG levels in untreated dogs were not associated with survival in multivariable analysis [34]. Although this was a smaller study and limited to a referral caseload, these differing findings to the current study may reflect intrinsic differences between typical treatment options and survival in a referral versus primary-care setting. In primary-care practice, early identification of DM, before BG levels rise > 40 mmol/L, may be important for this population of dogs to have a better chance of stabilisation and survival. Dogs starting insulin had a significantly reduced hazard of death (HR: 0.08, 95% CI 0.05–0.12, p < 0.001), consistent with the fact that virtually all dogs are insulin dependent by the time of diagnosis [9]. There was evidence from the current study that previous glucocorticoid treatment increased the hazard of death (HR: 1.83, 95% CI 1.20–2.80, *p* = 0.005). This may result from increased difficulties in managing DM concurrently with other disorders that require glucocorticoid treatment.

The presence of ketotis at diagnosis was not adversely associated with survival, and diabetic ketoacidosis was mentioned as a reason for euthanasia in only 3.4% of cases. Despite diabetic ketoacidosis potentially being a life-threatening condition, it appears not to be a driving factor for survival. This may reflect the confidence of primary practitioners in attempting treatment of the condition, rather than opting for euthanasia, or an under-reporting of the condition. The latter is likely to be true in this study where urine strips to identify ketones were used as a proxy for diabetic ketoacidosis at diagnosis, and where further investigations to diagnose diabetic ketoacidosis at the point of euthanasia was often not undertaken or reported in the clinical notes.

The study included information on a variety of management methods that were used to manage/monitor DM during the first 3 months following diagnosis, including BG curves at the practice, recommended diet change, spot BG and fructosamine measurements. At a univariable level, several of these methods were associated with increased survival. However, because these management methods were highly correlated with each other, and with insulin treatment, it was inappropriate to retain them all in the final model. Insulin treatment was retained as this had the greatest impact on the HR. Because dogs that survived longer had a longer duration of exposure to the chance of receiving management methods, it was aimed to minimise this survival-bias effect by restricting observations on management to the first 3 months post diagnosis. As many of the management methods were associated with increased survival, it can be argued that contact with the practice within these first few months is more important for survival than the type of management technique per se. This contact is likely to be a proxy for owner commitment and compliance, as well as the availability of support from the practice, and it has been argued that assessment of these on long term survival is more important than considering specific patient characteristics [49]. Further studies into survival of DM cases should consider ways to capture this element of owner compliance.

There were a number of limitations to this study. As it relied on reviewing of retrospective Electronic Patient Records (EPRs), inconsistencies and inaccuracies within these may have led to either missing data, or misclassification for variables such as obesity/medications/concurrent conditions. Errors relating to these inconsistencies were more likely to occur with controls than cases because the latter group were more likely to have higher counts of veterinary visits and investigations for concurrent conditions. The number of dogs within each breed category was dependant on the popularity of that breed within the UK within 2016. This meant there was insufficient power to detect potentially significant differences within breeds where there were low numbers, for example the inability of this study to detect Samoyeds as high risk for developing DM. Additionally, although misclassification of DM was thought unlikely because this is a routine primary-care veterinary diagnosis, a veterinary diagnosis of pancreatitis or hyperadrenocorticism in DM cases may have been misclassified due to similarities in clinical signs or difficulties in interpreting diagnostic tests.

## Conclusions

Middle-aged dogs, neutered males, specific breeds, glucocorticoid treatment and concurrent hyperadrenocorticism or pancreatitis were all associated with DM diagnosis in dogs. Additionally, there is an indication for further exploration of the associations between DM and obesity. Survival with DM is associated with age, breed and neutering. Survival was also associated with insulin treatment and BG level at diagnosis, suggesting early identification and treatment of DM patients in primary-care practice can help increase survival times. Awareness of these factors can aid veterinary professionals in the management and advice on prognosis after diagnosis of DM in dogs.

## Methods

### Study design and period

This study aimed to estimate prevalence and incidence, and investigate risk factors for DM and the survival of DM cases in dogs. This study used data from Electronic Patient Records (EPRs) of dogs attending UK primary-care veterinary practices collaborating within the VetCompass Programme. The study population consisted of the cohort of all dogs ≥3 years on the 1st January 2016 that were under the care of a VetCompass practice as evidenced by having an EPR during 2016, or in both 2015 and 2017. This study population was restricted to dogs ≥3 years for both cases and controls in order to exclude the less typical juvenile-onset DM cases which are likely to differ in their DM aetiology.

A nested case-control study was used to investigate risk factors for the diagnosis of DM, and the underlying study cohort was used to estimate prevalence, incidence and to examine survival post diagnosis. Sample size calculations estimated an unmatched case-control study would require approximately 350 cases and 700 controls to detect an odds ratio (OR) of at least 2.0 where at least 5% of the controls were exposed to the risk factor of interest (95% confidence level, 80% power, 1:2 case:control ratio) (OpenEpi v.3.012013). Ethical approval was granted by Royal Veterinary College Clinical Research Ethical Review Board (reference: SR2018–1652).

### Data collection and management

Potential DM cases were identified by searching the EPRs for terms related to diabetes diagnosis/management within the free text clinical records (*diab, insul, hyperg, mell, glucose, DM, ketoa, ketou, IDDM, fruct, curve, insuv, prozi, canins, vetp, vet pen*), treatment records (*canins, insul, prozi, neutral, lent, vetp, vet pen, insuv*), and recorded VeNom diagnosis fields (*Diabetes mellitus, Diabetes mellitus – unstable, Diabetes mellitus – stable, Diabetic ketoacidosis*). The EPRs were examined manually to identify cases, and their date of diagnosis. A DM case was defined as a final veterinary diagnosis of DM, treatment with insulin, or strong evidence of a veterinary diagnosis based on evidence of hyperglycaemia, glucosuria and appropriate clinical signs in the EPR. The date of diagnosis was the earliest date of either veterinary confirmation of diagnosis, receipt of confirmatory test results, or initiation of insulin treatment. Dogs treated with insulin for hyperkalaemia without hyperglycaemia were excluded. Cases that were pre-existing or newly diagnosed during 2016 were used to estimate a one-year period prevalence. Only cases newly diagnosed during 2016 were retained for estimation of incidence and for the risk factor and survival analysis.

### Case-control study

For each case, there were two control dogs randomly selected from the study population aged ≥3 years on 01/01/2016. Controls were excluded if the dog had pre-existing DM, developed DM prior to July 2019, or if the VetCompass record did not include evidence of direct owner/patient contact with the practice. To establish an equivalent to “date of diagnosis” for the controls, a random date in 2016 was generated for each dog and the nearest VetCompass record to this date was used to determine time-bound exposure variables.

Case and control demographic data extracted from VetCompass included: breed, date of birth, sex, neutering status, bodyweight, insurance status and veterinary group. Sex and neuter status were examined individually and also combined as a sex-neuter variable. Breeds were categorised as purebred or crossbred. Purebreds were further classified using the VeNom breed terms and analysed individually if they included ≥10 dogs and/or ≥ 5 DM cases in the overall case-control study. All purebred breeds containing < 10 dogs and/or < 5 DM cases were classified as purebred (other). Age was determined at the “date of diagnosis” and categorised into quartiles that grossly corresponded to 3 to < 8, 8 to < 10, 10 to < 13, and > 13 years. Adult mean bodyweight was calculated from all bodyweight data recorded from 18 months of age for each dog and used to classify dogs as at/above or below their breed/sex mean value. Veterinary group was categorised A – E; each group consisted of clinics that were part of the same parent company. The clinical records were examined manually to extract data on obesity, systemic glucocorticoid treatment within 6 weeks prior to diagnosis, or concurrent (+/− 3 months of diagnosis) diagnosis of pancreatitis or hyperadrenocorticism. Presence of obesity was recorded when there was evidence of a veterinary surgeon/nurse classifying the dog as very overweight, obese, or BCS ≥ 4/5 or ≥ 8/10, either at diagnosis or within the preceding 12 months. Evidence of pancreatitis was determined by a final veterinary diagnosis of pancreatitis, or a canine pancreatic lipase immunoreactivity of ≥400 μg/l. A diagnosis of hyperadrenocorticism included any dog initiated on trilostane treatment. Evidence for these latter two conditions was extended to +/− 3 months of diagnosis to account for similarities to DM in presenting signs, and unreliability of some hyperadrenocorticism tests in unstable diabetic dogs [50].

### Survival analysis

Survival analysis additionally included variables describing which diagnostic tests were used (urinalysis, BG, fructosamine, unspecified blood tests), BG level at diagnosis (as determined by a handheld glucometer, inhouse blood analyser, or via an external laboratory), evidence of ketosis at diagnosis (defined as a ketone recording of ≥1 on a urinalysis strip at or within 3 days of presentation, and prior to initiating insulin treatment), presence of cataracts within 3 months of diagnosis, referral for advanced clinical management, hospitalisation, initial insulin treatment regime, and management methods for the first 3 months (home BG measurements, BG curve, fructosamine, spot BG – defined as no more than one reading taken in 24 h, unspecified blood tests, urinalysis, recommended diet change). BG level at diagnosis was split primarily around the median and then 4 categories were created to reflect divisions of BG from above the renal threshold value (approx. 11.1 mmol/L) to the upper limit detected by handheld blood glucometers (approx. 40 mmol/L). These categories correspond to < 20 mmol/L, 20–30 mmol/L, 30–40 mmol/L, and > 40 mmol/L. Information on the date and cause of all deaths prior to February 2020 was extracted from the EPR. Records not explicitly recording death were censored at the date of the last practice-patient interaction prior to February 2020. Diabetes mellitus was recorded as the cause of death for deaths that occurred subsequent to the worsening of clinical signs. Euthanised cases were further classified as condition worsening, inability to cope with condition (owner), financial considerations, concurrent conditions or other. Data entry errors were minimised by a secondary coder checking every case and 15% of controls.

### Statistical analysis

Data were checked and cleaned in Microsoft Excel before exporting to Stata for analysis. One-year period prevalence was calculated as the number of newly diagnosed and pre-existing DM cases present in 2016 within the study population. Annual incidence risk with 95% confidence interval (CI) was calculated as the proportion of newly diagnosed DM cases in 2016 within the study population of dogs ≥3 years old on 01/01/2016.

The case-control study used univariable binary logistic regression modelling to explore associations between potential risk factors and the development of DM. Variables with a likelihood ratio test (LRT) of *p* < 0.2 were taken forward to multivariable analysis. Where checks for multicollinearity identifies an association, the variable with the lowest *p*-value within the multivariable model was retained. A manual forward selection method was used to build the final multivariable logistic regression model, retaining variables with a likelihood ratio test of *P* < 0.05. Final model variables were assessed for pairwise interaction effects using the LRT, confounding was assessed by evaluating for a > 10% change in parameter estimates, and all confounders were included in the final model. Clustering at the veterinary practice level was evaluated by including clinic ID as a random effect. Model fit was assessed using the Hosmer-Lemeshow test statistic [51], and statistical significance was set at 5%.

Median survival time was calculated for all-cause mortality, defined as the time from DM diagnosis until the cumulative survival proportion reached 50% [52]. Median survival time was calculated for all dogs, and also for a subset of dogs that survived at least day 7 following diagnosis. Cox regression modelling was used to assess associations with survival for all dogs. Variables associated with survival (*P* < 0.2) in the univariable analysis were taken forward to the multivariable analysis. Multivariable modelling used the same criteria as the logistic regression. Clinic ID was included as a frailty term to evaluate clustering at the practice level. The proportional hazards assumption was evaluated with Schoenfeld residuals and visual inspection of the Nelson-Aalen cumulative hazard plot. Statistical significance was set at 5%.

## Data Availability

The VetCompass™ dataset used for this study are available open access on the RVC data repository, http://researchonline.rvc.ac.uk/id/eprint/12622/

## Abbreviations

DM: Diabetes mellitus
WHWT: West Highland White Terrier
SBT: Staffordshire Bull Terrier
GSD: German Shepherd Dog
BG: Blood glucose
CI: Confidence interval
OR: Odds ratio
T1DM: Human Type 1 diabetes mellitus
HR: Hazard ratio
T2DM: Human Type 2 diabetes mellitus
MST: Median survival time
LRT: Likelihood ratio test
